# P-108 - HEALTH INEQUALITIES IN HEALTH PROTECTION IN NORTH LONDON, AN EXPLORATORY ANALYSIS

**DOI:** 10.1093/pubmed/fdag046.099

**Published:** 2026-07-09

**Authors:** Miss Hannah Walke, Dr Isabelle Whelan, Dr Alicia Thornton

**Affiliations:** UK Health Security Agency; UK Health Security Agency; UK Health Security Agency

## Abstract

**Background:**

Health Protection Teams (HPTs) should ensure their services do not create, or exacerbate existing, inequalities. Index of Multiple Deprivation (IMD) is a measure of local relative deprivation in England.

**Objectives:**

This analysis aimed to explore HPT casework with a focus on health equity, using IMD as a measure of deprivation.

**Methods:**

Confirmed or probable cases notified in 2025 and resident in North London were extracted from the Case and Incident Management System (CIMS). Cases were assigned an IMD 2019 decile based on residential postcode and analysed in two groups: a) all cases notified and b) acute cases managed by the clinical team. R was used to analyse the number of cases in each decile and the rate per 100,000 population (based on total population of all Lower Super Output Areas in that decile).

**Results:**

For acute (n = 2525) and all cases (n = 15983), IMD decile 3 had the highest number of cases notified. In general, the number of cases notified decreased as deprivation decreased. Gastrointestinal infections, vaccine-preventable disease and tuberculosis provided the greatest number of acute cases. IMD decile 1 had the highest rate of cases notified per 100,000 population, which decreased towards the least deprived deciles, when analysed for both acute and all case notifications. Ethnicity was recorded for only 38% (965/2525) of acute cases.

**Conclusions:**

In the North London population, those living in more deprived areas experience a higher burden of reported infectious disease. This analysis did not include further quantification of inclusion health characteristics, as they are not routinely recorded in the case management system; a limitation of CIMS. There is a need to improve the recording of health equity parameters within CIMS, in particular ethnicity and information about specific risk groups, so that HPTs can appropriately assess their impact on health inequalities.

